# The Frequency of Left Atrial Thrombus on Transthoracic Echocardiogram in Patients with Mitral Stenosis

**DOI:** 10.7759/cureus.7453

**Published:** 2020-03-29

**Authors:** Khalil Ahmed, Aziz Rehman Memon, Hussain Liaquat, Mustajab Mujtaba, Chander Parkash, Fateh Ali Tipoo Sultan, Musa Karim

**Affiliations:** 1 Cardiology, National Institute of Cardiovascular Diseases (NICVD), Karachi, PAK; 2 Critical Care Medicine, National Institute of Cardiovascular Diseases (NICVD), Karachi, PAK; 3 Cardiology, Aga Khan University Hospital, Karachi, PAK; 4 Statistics, National Institute of Cardiovascular Diseases (NICVD), Karachi, PAK

**Keywords:** left atrial thrombus, mitral stenosis, transthoracic echocardiogram

## Abstract

Background

Patients with mitral stenosis (MS) are more prone to develop left atrial (LA) thrombus. This cross-sectional study was conducted to determine the frequency of LA thrombus on transthoracic echocardiography (TTE) in patients with MS.

Methods

In this study, we included patients diagnosed with MS undergoing TTE at the echocardiography department of the National Institute of Cardiovascular Disease (NICVD), Karachi, Pakistan. The severity of MS was classified based on the mitral valve area (MVA) as follows: very severe: MVA of ≤1.0 cm^2^; severe: MVA of ≤1.5 cm^2^; and mild to moderate: MVA of >1.5 cm^2^. The LA thrombus was observed and noted on TTE.

Results

A total of 256 MS patients were included in this study, out of which 46.5% (119) were male. The mean age was 33.78 ±11.51 years. MS was classified as mild to moderate in 3.5% of the patients, severe in 54.3%, and very severe in 42.2%. In 98.8% of the patients, the etiology of MS was rheumatic. LA thrombus was observed in 25% (64) of the patients and LA smoke was observed in 12.1% (31). Among other findings, mitral regurgitation (MR) was observed in 17.2% of the patients, aortic regurgitation (AR) in 5.1%, aortic stenosis (AS) in 4.7%, and tricuspid regurgitation (TR) in 48.8%. Five (2%) patients had atrial septal defect (ASD), 17.3% had left ventricular (LV) dysfunction, 15.2% had right ventricular (RV) dysfunction, and vegetation was seen in 11.8% of the patients. Patients with LA thrombus were found to be associated with the following conditions on a higher scale compared to those without: decreased ejection fraction (EF) (52 ±8.5% vs. 54.94 ±6.6%; p: 0.011); RV dysfunction (39.1% vs. 7.3%; p: <0.001); and presence of associated pathologies (82.8% vs. 43.8%; p: <0.001).

Conclusion

LA thrombus on TTE was detected in a significant number (25%) of patients with MS. It was also found to be strongly associated with the severity of the disease, reduced EF, RV dysfunction, and the presence of associated value pathologies.

## Introduction

A significant reduction was witnessed in the prevalence, incidence, and associated fatalities of rheumatic heart disease (RHD) in high-income countries in the late 20th century [1]. Containment of this fatal, yet preventable, cardiovascular disease can be partly attributed to improved living standards and widespread use of penicillin G benzathine for the treatment and prevention of rheumatic fever (RF) [2,3]. Age-standardized global mortality due to RHD reportedly declined by 47.8% between the years 1990 and 2015 [1]. However, it still remains one of the major causes of death and disability-adjusted life-years in the low- and middle-income countries (LMIC) [4,5]. The surging burden of RHD in LMIC can be attributed to poor living conditions, poor sanitation, overcrowdedness, and inadequate healthcare infrastructure [6,7].

Mitral stenosis (MS) is the most common among the long-term complications of RF in adults, but it can be congenital in infants too [8]. Patients with MS, especially those with atrial fibrillation, are more prone to left atrial (LA) thrombus formation [9,10]. The changed anatomy and enlarged volume of LA, abnormal blood flow into the atrium, abnormal atrial contraction, decreased mitral valve area (MVA), and impaired endothelial function are the key determinants of increased risk of thrombus formation in these patients [11]. The presence of LA thrombus is associated with a threefold increase in the risk of embolic events [12,13]. Along with the severity of MS, various other predisposing factors for LA thrombus have been described in various studies, such as advanced age, duration of symptoms, low cardiac output state, and LA size [14-16].

Although contrast-enhanced MRI scans provide high sensitivity and specificity for the detection of LA thrombus, due to widespread availability and easy adaptability, echocardiography is now considered the best available non-invasive modality for its detection [17]. The accuracy of echocardiography, more specifically transthoracic echocardiography (TTE), is disputed, especially in cases where the thrombus is formed in LA appendage. In such cases, transesophageal echocardiography (TEE) has shown higher accuracy and sensitivity [18].

Due to a lack of education, actability, recourses, and awareness about the disease, the majority of patients in our population tend to present late for treatment, and usually with very severe conditions. As a consequence, even TTE is able to detect LA thrombus with high accuracy in such patients. This study was designed to quantify the frequency of LA thrombus on TTE in patients with MS at a tertiary care cardiac center in Karachi, Pakistan.

## Materials and methods

In this observational study, we included patients undergoing TTE at the echocardiography department of the National Institute of Cardiovascular Disease (NICVD), Karachi, Pakistan from September 2018 to January 2019. Inclusion criteria were as follows: both male and female patients between 15-65 years of age who are diagnosed with MS. Patients with a history of any prior cardiac surgery or those who were currently on anticoagulation therapy were excluded. Informed consent was obtained for the TTE along with consent regarding the inclusion of patients in the study and to use obtained data for research and publications while maintaining the anonymity of the patient.

The TTE studies were performed by consultant echocardiographers by a standard technique, and echocardiographic parameters such as MVA, LA size, left ventricular diastolic diameter (LVDD), left ventricular systolic diameter (LSDD), and aorta diameter were measured as per the American Society of Echocardiography criteria [19]. MS was classified as very severe if MVA was ≤1.0 cm^2^, severe if MVA was ≤1.5 cm^2^, and mild to moderate if MVA was >1.5 cm^2^. The etiology of MS was considered rheumatic when commissural fusion and diastolic doming of the mitral valve leaflets were seen. The LA thrombus was observed and presence was noted on TTE. To attain the maximum visualization of thrombus, LA was examined in multiple transthoracic views with angulation of transducer to increase visualization of LA appendage, such as standard parasternal long axis, subcostal view, apical view, and parasternal short-axis view.

The presence and severity of the associated valve pathologies such as mitral regurgitation (MR), aortic regurgitation (AR), aortic stenosis (AS), tricuspid regurgitation (TR), and atrial septal defect (ASD) along with pulmonary artery pressure, ejection fraction (EF), right ventricular (RV) dysfunction, and presence of vegetation were also recorded. Obtained echocardiographic data along with patients demographics, such as age and gender, were recorded on a proforma and entered on to a data entry screen designed using the Census and Survey Processing System (CSPro) version 7.0. Data analysis processes were carried out using IBM SPSS, Version 21.0. (IBM Corp., Armonk, NY). Mean ±standard deviation (SD) and frequency were calculated for continuous and categorical response data respectively. Patients were stratified based on the presence of LA thrombus, and the appropriate Chi-square test and independent-sample Student's t-test were performed. A two-sided p-value of ≤0.05 was considered as statistically significant.

## Results

This observational study included a total of 256 patients with MS, out of which 53.5% (137) were female. The mean age of the cohort was 33.78 ±11.51 years. A significant number of patients [42.2% (108)] had very severe MS and pathophysiology of MS was rheumatic in 98.8% (253) of the patients. Thrombus in LA on TTE was observed in 25% (64) of patients and 12.1% (31) patients had LA smoke. The presence of LA thrombus was found to be significantly associated with the severity of MS (p: <0.001), which was observed with the frequency of 0% (0/9), 8.6% (12/139), and 48.1% (52/108) in patients with mild to moderate, severe, and very severe MS respectively. Gender and age distribution and TTE findings in the study population are presented in Table 1.

**Table 1:** Gender and age distribution and transthoracic echocardiography findings in the study population SD: standard deviation; MS: mitral stenosis; LA: left atrial; LVDD: left ventricular diastolic diameter; LSDD: left ventricular systolic diameter

Characteristics	Values
Total	256
Gender, n (%)	
Male	119 (46.5)
Female	137 (53.5)
Age, years, mean ±SD	33.78 ±11.51
Up to 40 years, n (%)	197 (77)
41-65 years, n (%)	56 (21.9)
>65 years, n (%)	3 (1.2)
Severity of MS, n (%)	
Mild to moderate	9 (3.5)
Severe	139 (54.3)
Very severe	108 (42.2)
LA thrombus, n (%)	64 (25)
LA smoke, n (%)	31 (12.1)
Echocardiographic parameters, mean ±SD, mm	
LA size	45.25 ±7.98
LVDD	40.46 ±5.16
LSDD	27.82 ±5.58
Aorta diameter	31.29 ±5.61

Among other findings, MR was observed in 17.2% of the patients, AR in 5.1%, AS in 4.7%, and TR in 48.8%. Five (2%) patients had ASD, 17.3% had LV dysfunction, 15.2% had RV dysfunction, and vegetation was seen in 11.8% of the patients. Patients with LA thrombus were found to be associated with the following conditions on a higher scale compared to those without: decreased EF (52 ±8.5% vs. 54.94 ±6.6%; p: 0.011); RV dysfunction (39.1% vs. 7.3%; p: <0.001); and the presence of associated pathologies (82.8% vs. 43.8%; p: <0.001). TTE parameters and associated valve pathologies stratified by the presence of LA thrombus in the study population are presented in Table 2.

**Table 2:** Transthoracic echocardiography parameters and associated valve pathology stratified by the presence of left atrial thrombus in the study population LA: left atrial; MR: mitral regurgitation; AR: atrial regurgitation; AS: atrial stenosis; TR: tricuspid regurgitation; ASD: atrial septal defect; SD: standard deviation; LV: left ventricular; EF: ejection fraction; RV: right ventricular

Pathologies and defects	Values	LA thrombus		P-value
		Present	Absent	
Total patients	256	64	192	-
Associated valve pathologies, n (%)	137 (53.5)	53 (82.8)	84 (43.8)	<0.001
MR, n (%)	44 (17.2)	16 (25)	28 (14.6)	0.056
Mild, n (%)	28 (63.6)	10 (62.5)	18 (64.3)	0.906
Moderate, n (%)	16 (36.4)	6 (37.5)	10 (35.7)	
Severe, n (%)	0 (0)	0 (0)	0 (0)	
AR, n (%)	13 (5.1)	3 (4.7)	10 (5.2)	0.869
Mild, n (%)	12 (92.3)	3 (100)	9 (90)	0.569
Moderate, n (%)	1 (7.7)	0 (0)	1 (10)	
Severe, n (%)	0 (0)	0 (0)	0 (0)	
AS, n (%)	12 (4.7)	5 (7.8)	7 (3.6)	0.172
Mild, n (%)	6 (50)	5 (100)	1 (14.3)	0.014
Moderate, n (%)	3 (25)	0 (0)	3 (42.9)	
Severe, n (%)	3 (25)	0 (0)	3 (42.9)	
TR, n (%)	125 (48.8)	49 (76.6)	76 (39.6)	<0.001
Mild, n (%)	37 (29.6)	12 (24.5)	25 (32.9)	0.603
Moderate, n (%)	62 (49.6)	26 (53.1)	36 (47.4)	
Severe, n (%)	26 (20.8)	11 (22.4)	15 (19.7)	
ASD, n (%)	5 (2)	0 (0)	5 (2.6)	0.192
Pulmonary artery pressure, mean ±SD, mmHg	58.72 ±16.2	62.39 ±17.7	57.47 ±15.6	0.083
LV dysfunction, n (%)	44 (17.3)	19 (29.7)	25 (13.1)	0.192
Decreased EF, mean ±SD, %	54.19 ±7.2	52 ±8.5	54.94 ±6.6	0.011
RV dysfunction, n (%)	39 (15.2)	25 (39.1)	14 (7.3)	<0.001
Mild, n (%)	23 (59)	13 (52)	10 (71.4)	0.237
Moderate, n (%)	16 (41)	12 (48)	4 (28.6)	
Severe, n (%)	0 (0)	0 (0)	0 (0)	
Vegetation, n (%)	30 (11.8)	7 (10.9)	23 (12.1)	0.802
Anterior mitral leaflet, n (%)	22 (73.3)	7 (100)	15 (65.2)	0.19
Posterior mitral leaflet, n (%)	6 (20)	0 (0)	6 (26.1)	
Others, n (%)	2 (6.7)	0 (0)	2 (8.7)	

## Discussion

LA thrombus in patients with MS is a challenging complication associated with the increased risk of systemic embolization resulting in higher mortality and morbidity [20,21]. Even though TEE is the well-established gold standard for the detection of LA thrombus, in our clinical observation, the majority of patients presented with very severe conditions due to late presentation, lack of awareness, actability, and limitation of recourses, and hence, even TTE was able to detect LA thrombus with high accuracy in our population. In this selected cohort, we observed that a significant number of patients presented with very severe MS (42.2%), and LA thrombus on TTE was detected in 25%, which is much higher than the reported frequency of 3.8-22.8% in some of the previous regional studies [16,20,22-24]. The pertinent reason for the higher frequency of LA thrombus in our study could be the higher proportion of patients with very severe MS. This is evident from our results as they indicated that the severity of diseases is strongly associated with the presence of thrombus. Also, the association between the severity of MS and the presence of LA thrombus was indeed reported in one past study [20].

Most of the studies conducted for this population are based on TEE [20,23,24]. Although the sensitivity and specificity of TTE are disputed, the detection of LA thrombus using LA enlargement of TTE is reported to be nearly 90% accurate, with a sensitivity of 88.9% and specificity of 52.2%, based on a study of patients with ischemic stroke [25]. Atrial fibrillation, smaller LA size, bigger MVA, and no low-flow velocities are also reported to be associated with LA thrombus [20,23]. In our study, associated valve pathologies such as TR, RV dysfunction, and reduced EF were found to be associated with LA thrombus.

In the era of percutaneous interventions, a clot in LA is one of the contraindications for percutaneous balloon mitral valvuloplasty (PBMV) due to the risk of embolism [24]. Guidelines recommend vitamin K antagonists (VKA) such as warfarin or novel anticoagulants for the resolution of thrombus [26]. Studies have shown the resolution of thrombus after short- and long-term (three and six months) of anticoagulation [27-29].

A pertinent lack of accuracy, sensitivity, and specificity of TTE in the detection of LA thrombus and the non-availability of TEE studies due to lack of resources and time are the key limitations of this study. TTE can be a good screening modality for the assessment of LA thrombus in resource-limited settings; however, for clinical decision-making for management options, TEE confirmation should indeed be considered.

## Conclusions

Our study was designed to quantify the frequency of LA thrombus on TTE in patients with MS. In our study, LA thrombus on TTE was detected in a significant number (25%) of the patients with MS. Additionally, it was found to be strongly associated with the severity of the disease, decreased EF, RV dysfunction, and the presence of associated value pathologies.

## References

[1] Watkins DA, Johnson CO, Colquhoun SM (2017). Global, regional, and national burden of rheumatic heart disease, 1990-2015. N Engl J Med.

[2] Gordis L (1985). The virtual disappearance of rheumatic fever in the United States: lessons in the rise and fall of disease. T. Duckett Jones memorial lecture. Circulation.

[3] Massell BF, Chute CG, Walker AM, Kurland GS (1988). Penicillin and the marked decrease in morbidity and mortality from rheumatic fever in the United States. N Engl J Med.

[4] Doukky R, Abusin SA, Bayissa YA, Kelly RF, Ansari AH (2014). Rheumatic heart disease in modern urban America: a cohort study of immigrant and indigenous patients in Chicago. Int J Cardiol.

[5] Rothenbühler M, O'Sullivan CJ, Stortecky S (2020). Active surveillance for rheumatic heart disease in endemic regions: a systematic review and meta-analysis of prevalence among children and adolescents. Lancet Glob Health.

[6] Marijon E, Mirabel M, Celermajer DS, Jouven X (2012). Rheumatic heart disease. Lancet.

[7] Meira ZM, Goulart EM, Colosimo EA, Mota CC (2005). Long term follow up of rheumatic fever and predictors of severe rheumatic valvar disease in Brazilian children and adolescents. Heart.

[8] Olson LJ, Subramanian R, Ackermann DM, Orszulak TA, Edwards WD (1987). Surgical pathology of the mitral valve: a study of 712 cases spanning 21 years. Mayo Clin Proc.

[9] Belen E, Özal E, Püsüroğlu H (2016). Relationship between the presence of left atrial thrombus in patients with mitral stenosis and platelet-to-lymphocyte ratio. Anatol J Cardiol.

[10] İşcan Ş, Dönmez K, Çakır H, Kestelli M (2016). LA thrombus formation in mitral valve disease. Anatol J Cardiol.

[11] Watson T, Shantsila E, Lip GY (2009). Mechanisms of thrombogenesis in atrial fibrillation: Virchow's triad revisited. Lancet.

[12] Kronzon I, Tunick PA, Charney LH (2006). Echocardiography as a tool in the evaluation of conditions with a high likelihood of cardiogenic embolism. Isr Med Assoc J.

[13] Acartürk E, Usal A, Demir M, Akgül F, Ozeren A (1997). Thromboembolism risk in patients with mitral stenosis. Jpn Heart J.

[14] Agarwal AK, Venugopalan P (2001). Left atrial spontaneous echo contrast in patients with rheumatic mitral valve stenosis in sinus rhythm: relationship to mitral valve and left atrial measurements. Int J Cardiol.

[15] Goswami KC, Yadav R, Rao MB, Bahl VK, Talwar KK, Manchanda SC (2000). Clinical and echocardiographic predictors of left atrial clot and spontaneous echo contrast in patients with severe rheumatic mitral stenosis: a prospective study in 200 patients by transesophageal echocardiography. Int J Cardiol.

[16] Fazlinezhad A, Golmohammadzadeh H, Azari A, Bigdelu L (2014). Echocardiographic predictors of left atrial thrombus in patients with severe rheumatismal mitral stenosis. Razavi Int J Med.

[17] Srichai MB, Junor C, Rodriguez LL (2006). Clinical, imaging, and pathological characteristics of left ventricular thrombus: a comparison of contrast-enhanced magnetic resonance imaging, transthoracic echocardiography, and transesophageal echocardiography with surgical or pathological validation. Am Heart J.

[18] Manjunath CN, Srinivasa KHS, Panneerselvam A, Prabhavathi B, Ravindranath KS, Rangan K, Dhanalakshmi C (2011). Incidence and predictors of left atrial thrombus in patients with rheumatic mitral stenosis and sinus rhythm: a transesophageal echocardiographic study. Echocardiography.

[19] Wiegers SE, Ryan T, Arrighi JA (2019). 2019 ACC/AHA/ASE advanced training statement on echocardiography (revision of the 2003 ACC/AHA clinical competence statement on echocardiography): a report of the ACC Competency Management Committee. J Am Coll Cardiol.

[20] Bajwa A, Hyder SN, Aziz Z (2016). Echocardiographic predictors of left atrial thrombus formation in patients with rheumatic mitral stenosis. Pak Heart J.

[21] Shah SD, Bari SA, Ali H, Kasi MZ (2018). Frequency of left atrial thrombus in patients of mitral stenosis with atrial fibrillation. Pak Heart J.

[22] Saidi SJ, Motamedi MH (2004). Incidence and factors influencing left atrial clot in patients with mitral stenosis and normal sinus rhythm. Heart.

[23] Mahmood ul Hassan, Hussain C, Gul AM, ullah Jan H, Hafizullah M (2010). Frequency of left atrial and appendage clot in patients with severe mitral stenosis. J Ayub Med Coll Abbottabad.

[24] Gill BUA, Abbas T, Haq RU, Qureshi BA, Hashmi KA, Ahmed I, Javaid A (2015). Frequency of left atrial thrombus in patients of mitral stenosis suitable for percutaneous trans-septal mitral commissurotomy. Pak Heart J.

[25] Anaissie J, Monlezun D, Seelochan A (2016). Left atrial enlargement on transthoracic echocardiography predicts left atrial thrombus on transesophageal echocardiography in ischemic stroke patients. Biomed Res Int.

[26] Kim WJ, Park JM, Kang K (2016). Adherence to guidelines for antithrombotic therapy in patients with atrial fibrillation according to CHADS2 score before and after stroke: a multicenter observational study from Korea. J Clin Neurol.

[27] Lee WC, Fang CY, Chen YL (2019). Left atrial or left atrial appendage thrombus resolution after adjustment of oral anticoagulant treatment. J Stroke Cerebrovasc Dis.

[28] Silaruks S, Thinkhamrop B, Kiatchoosakun S, Wongvipaporn C, Tatsanavivat P (2004). Resolution of left atrial thrombus after 6 months of anticoagulation in candidates for percutaneous transvenous mitral commissurotomy. Ann Intern Med.

[29] Srimannarayana J, Varma RS, Satheesh S, Anilkumar R, Balachander J (2003). Prevalence of left atrial thrombus in rheumatic mitral stenosis with atrial fibrillation and its response to anticoagulation: a transesophageal echocardiographic study. Indian Heart J.

